# Large-scale prediction of protein-protein interactions from structures

**DOI:** 10.1186/1471-2105-11-144

**Published:** 2010-03-18

**Authors:** Martial Hue, Michael Riffle, Jean-Philippe Vert, William S Noble

**Affiliations:** 1 Mines ParisTech, Centre for Computational Biology, 35 rue Saint-Honoré, F-77305 Fontainebleau, France; 2 Institut Curie, F-75248, Paris, France; 3 INSERM U900, F-75248, Paris, France; 4 Department of Biochemistry, University of Washington, Seattle, WA, USA; 5 Department of Genome Sciences University of Washington, Seattle, WA, USA; 6 Department of Computer Science and Engineering, University of Washington, Seattle, WA, USA

## Abstract

**Background:**

The prediction of protein-protein interactions is an important step toward the elucidation of protein functions and the understanding of the molecular mechanisms inside the cell. While experimental methods for identifying these interactions remain costly and often noisy, the increasing quantity of solved 3D protein structures suggests that *in silico *methods to predict interactions between two protein structures will play an increasingly important role in screening candidate interacting pairs. Approaches using the knowledge of the structure are presumably more accurate than those based on sequence only. Approaches based on docking protein structures solve a variant of this problem, but these methods remain very computationally intensive and will not scale in the near future to the detection of interactions at the level of an interactome, involving millions of candidate pairs of proteins.

**Results:**

Here, we describe a computational method to predict efficiently *in silico *whether two protein structures interact. This yes/no question is presumably easier to answer than the standard protein docking question, "How do these two protein structures interact?" Our approach is to discriminate between interacting and non-interacting protein pairs using a statistical pattern recognition method known as a support vector machine (SVM). We demonstrate that our structure-based method performs well on this task and scales well to the size of an interactome.

**Conclusions:**

The use of structure information for the prediction of protein interaction yields significantly better performance than other sequence-based methods. Among structure-based classifiers, the SVM algorithm, combined with the metric learning pairwise kernel and the MAMMOTH kernel, performs best in our experiments.

## Background

The knowledge of the interactions among proteins is essential to understanding the molecular mechanisms inside the cell. However, the experimental measurement of protein-protein interactions by two main procedures--the yeast two-hybrid system and mass spectrometry combined with tandem affinity purification--suffers from a high false positive rate, as evidenced by the small intersection between several independently generated interaction data sets [1]. Recent years have seen much progress in understanding of the false positive predictions [2]. The limitations of current experimental methods therefore highlight the need for *in silico *interaction predictions.

The elucidation of an increasing number of protein 3D structures is likely to continue at a fast pace as a result of several large-scale initiatives. These structures provide both an opportunity and a challenge for *in silico *prediction methods. The opportunity is that if *in silico *methods are able to predict whether two given 3D structures interact, then these methods may be applied to predict interactions among the large amount of proteins with known or inferred 3D structure. The challenge is that these methods need to be computationally fast to scale to the prediction of interactions among millions or more candidate pairs of structures. Unfortunately, the current methods of choice to predict interactions are mostly based on the idea of docking, which is very computationally intensive and therefore unlikely to be able to scale to large interactomes in the near future. See [3] for a review of the issues related to the prediction of interaction using protein-protein docking, and [4] for a review of the problem of predicting interactions using structural information.

In this study, we are interested in developing methods to predict *whether *two proteins interact. We aim to develop a method that will scale to the analysis of whole proteomes in order to identify candidate protein pairs for further analysis by more computationally expensive procedures. Docking not only attempts to answer the question of whether two proteins interact, but also *how *they interact. Our strategy is to develop methods that predict less information than docking, by focusing only on the first question, trading this decrease in the richness of prediction for an increase in computational efficiency. Note that, in general, the problem of predicting protein-protein interactions is complicated by multi-domain proteins. For simplicity in this work, we will therefore focus on predicting whether a given pair of protein domains interacts. We aim at answering the question: "Given two domain structures, do they interact?" Our strategy to solve this problem is to formalize it as a binary classification problem, and to train a discriminative classifier to answer the question of whether or not a pair of domain structures interact, using as training data examples of known interacting and non-interacting pairs.

This idea is related to previous applications of machine learning techniques to predict protein-protein interactions from a variety of data types, including noisy interaction networks, localization information, sequence and expression data. These techniques include Bayesian networks [5], support vector machines [6-9], decision trees [10,11] and random forests [12]. Shoemaker *et al*. [13] review the approaches for predicting protein-protein interactions, and [14] compare existing approaches on a common data set. However, to our knowledge, machine learning methods have not previously been applied to the prediction of protein-protein interactions from protein structures.

¿From a technical point of view, we face two issues to implement this strategy. First, we must be able to manipulate protein 3D structures in the context of a machine learning algorithm. For this we borrow an idea from [15], who proposed using a classical measure of structural similarity to implicitly embed 3D structures in a vector space. Second, once we represent each 3D structure as a vector, we must be able to learn a classification function over *pairs *of vectors. For this we test different strategies that have been proposed recently to solve this issue with kernel methods such as SVMs [9,16]. Combining these two ideas, we propose two learning algorithms that can be combined with two possible representations of pairs of 3D structures to predict interactions, resulting in four methods. In order to assess the benefits of using 3D structure information for this purpose, rather than using sequence information only, we additionally test the same four methods starting from a measure of primary sequence similarity, instead of 3D structure similarity.

In order to test these methods, we construct three benchmarks from known interactions between proteins of known or inferred structures. We compare the performance of the different methods in a cross-validation procedure. As a baseline, we compare to similar methods that exploit only amino acid sequences. These experiments suggest that the metric learning pairwise kernel [16], coupled with a support vector machine classifier, yields the best performance.

## Methods

Our approach to predict whether two proteins interact is to frame the question as a supervised learning problem. Based on examples of interacting pairs and non-interacting pairs, we train a binary classifier to predict the class ("interacting" or "non-interacting") of a pair of protein structures. In this study, we compare eight different classification methods, corresponding to all combinations with respect to three binary choices: (1) two classification algorithms, (2) two vector encodings of proteins, based on structure or sequence, respectively, and (3) two different methods for computing the similarities among pairs of vectors. In the following, we first describe the two classification algorithms, the two similarity functions, and then the two methods for computing pairwise similarities.

### Classification algorithms

#### Nearest neighbor

As a simple baseline algorithm, we use the nearest neighbor algorithm (NN). Given a training set of *n *points *x*_1_,...,*x*_*n *_labeled *y*_1_,...,*y*_*n *_in {-1, +1}, NN assigns to a test point  the class label of the majority among the *k *nearest training set points. In our case, rather than a simple binary label, we would like the algorithm to return a real-valued discriminant score in order to be able to rank the prediction by confidence. For this purpose, we use the difference in the sum of the distances to the *k *nearest positive and the sum of distances to the *k *nearest negative training set examples:(1)

The predicted class label is simply the sign of this discriminant.  (resp. ) denotes the set of *k *nearest negative (resp. positive) neighbors. The value of *k *is selected via cross-validation, as described in the Results section.

NN is perhaps the simplest example of a broad class of algorithms known collectively as *kernel methods *[17]. An algorithm is a kernel method if and only if it can be formulated such that every occurrence of a data vector *x *occurs inside of a scalar product operation. In this case, every instance of the scalar product operation can be replaced by a generalized notion of similarity, called the *kernel function*. Formally, a kernel function is a symmetric positive semidefinite function, which provably corresponds to the scalar product operation in some vector space. Using a kernel function in place of the scalar product operation implicitly maps the given data set into a possibly high-dimensional space (called the *feature space*) that may be non-linearly related to the *input space *in which the data resides.

To "kernelize" the nearest neighbor algorithm, we simply note that the Euclidean distance can be stated entirely in terms of scalar product operations:

In terms of the kernel function *K*(, *x*) = ⟨;Φ(), Φ(*x*)⟩;, therefore,(2)

The kernel NN algorithm simply substitutes Equation (2) into Equation (1). Below, we define three different kernel functions and use each of them to create a NN classifier for predicting protein-protein interactions.

#### Support vector machine

Functionally, the support vector machine (SVM) [18] is similar to the nearest neighbor algorithm. Both are supervised classification algorithms, and both are kernel methods. However, unlike the nearest neighbor approach, the SVM algorithm attempts to find a hyperplane that optimally separates examples from the two given classes. The SVM algorithm is appealing due to its mathematical properties and its successful application to a wide variety of classification problems in computational biology [19].

Training an SVM involves solving a convex quadratic program, which can be formulated as follows:

subject to the constraints

The solution to this optimization is a hyperplane *h*_*w*,*b *_= {*x*: ⟨*w*, Φ(*x*)⟩ + *b *= 0}, and the classification procedure for a novel test point  involves computing which side of the hyperplane the point lies on. The SVM discriminant is the signed distance to the hyperplane:

Like NN, the SVM can also be subjected to the "kernel trick," the details of which we omit here for brevity. The SVM depends on a user-specified regularization term, *C *≥ 0, which prevents overfitting to noise in the data or noise in the labels. In the case of unbalanced data, we apply this regularization asymmetrically to positive and negative examples, as follows:(3)

The values of *C*^+ ^and *C*^- ^are selected via cross-validation, as described in the Results section.

### Kernel on proteins

In order to use a NN or SVM algorithm to predict interactions between proteins, we must define a kernel between the objects for which predictions are to be made, namely, pairs of proteins. In this section, we describe two kernels that are defined with respect to individual proteins. In the next section, we describe methods for generalizing from these single-protein kernels to kernels on protein pairs.

#### A kernel on protein structures

A natural place to begin, when considering comparisons among protein structures, is with structural alignment algorithms such as CE [20], DALI [21] and MAMMOTH [22]. These algorithms create an alignment between two proteins and then compute a score that reflects the alignment's quality. In this work, we use MAMMOTH [22], which is efficient and produces high quality alignments. We treat the output of MAMMOTH as an arbitrary score, denoted *s*(*p, q*).

Unfortunately, the alignment quality score returned by MAMMOTH cannot be used as a kernel function directly, because the score is not positive semidefinite (i.e., for a given set of protein structures, an all-versus-all matrix of MAMMOTH scores will usually have some negative eigenvalues). To define a MAMMOTH kernel on protein structures, we therefore subtract the negative portion of the eigenvalue spectrum to convert the score to a kernel. Namely, if *M *is the MAMMOTH similarity matrix, then *M *is symmetric with singular value decomposition

where *D *is a diagonal matrix diag(*λ*_1_,...,*λ*_*n*_). The MAMMOTH kernel is then

with Ψ(*D*) = diag(*ψ*(*λ*_1_),...,*ψ*(*λ*_*n*_)), and *ψ*(*λ*) = 1 + *λ *if *λ *> 0, and 0 otherwise.

In practice, we normalize this kernel by projecting onto the unit sphere, via the transformation(4)

A related MAMMOTH kernel has previously been shown to yield good performance in classifying proteins into SCOP categories or by GO terms [15]. It uses *ψ*(*λ*) = *λ*^2^.

#### A kernel on protein sequences

We compare our methods based on structures to corresponding sequence-based methods, as a baseline. We borrow an idea of a kernel on sequences from [23]. The mismatch kernel induces a distance measure between protein sequences. A protein sequence *α *is mapped to a feature vector

where  is the alphabet of 20 amino acids. The neighbourhood _*k*,*m*_(*α*) of a *k*-mer *α *is the set of *k*-mers that differs in at most *m *positions. The feature vector encodes the all the *k*-mers in the neighborhood:

Thus, the mismatch kernel between two protein sequences *x *and *y *is

In our experiments, we set *k *= 6 and *m *= 1.

### Kernels for pairs of objects

This section describes techniques for deriving, from the single-protein kernels described in the previous Section, a pairwise kernel function *K*((*p*_1_, *p*_2_), (*q*_1_, *q*_2_)) that quantifies the degree of similarity between two protein pairs. In particular, we present two pairwise kernels: the tensor product pairwise kernel (TPPK) and the metric learning pairwise kernel (MLPK).

#### Tensor product pairwise kernel

The tensor product pairwise kernel (TPPK) is a general method for building a kernel for pairs of objects from any kernel *K *for objects. This kernel has been used successfully for predicting protein-protein interactions from sequence [24] and from a combination of sequence, annotation, network topology and interolog features [9].

The TPPK considers two pairs of proteins to be similar to one another when each protein from one pair is similar to one protein of the other pair. For example, if *p*_1 _is similar to *q*_1 _and *p*_2 _is similar to *q*_2_, then we can say that the pairs (*p*_1_, *p*_2_) and (*q*_1_, *q*_2_) are similar to one another. We can translate these intuitions into the following pairwise kernel:(5)

where *K *is the kernel on proteins. The feature space for TPPK is the tensor product of the feature spaces of each member of the pair, so if *M *is the dimensionality of the feature space, then the dimensionality of the feature space of the tensor product is *M*^2^.

Note that, as formulated in Equation (5), the TPPK has the counter-intuitive property that two protein pairs can be considered similar if the underlying protein pairs are strongly dissimilar. This is because the base kernel function *K *can return negative values which, when multiplied together, yield a positive value for *K*_*TPPK*_. To avoid this artifact, we add 1 to the base kernel function, so that the kernel values lie in the range [0, 2]. This operation preserves a valid kernel, because the matrix of all 1's is positive definite, and the sum of two positive definite matrices is a positive definite matrix. Without this "add 1" correction, the TPPK results are significantly worse (data not shown).

#### Metric learning pairwise kernel

Similar to TPPK, the metric learning pairwise kernel (MLPK) is a method for building a kernel for pairs of objects based on a kernel *K *for objects [16]. However, MLPK represents a pair of objects as the difference between its members. In this case, a pair (*p*_1_, *p*_2_) of objects is represented in the feature space by the difference *p*_1 _- *p*_2_, squared (by the tensor product operation) to make it invariant with respect to the order of the proteins. The MLPK was presented in [16] as a way to learn a new metric between individual objects (3D structures in our case). The corresponding MLPK between the pair (*p*_1_, *p*_2_) and (*q*_1_, *q*_2_) is(6)

For consistency with the TPPK, we add 1 to each entry of *K*.

### Data

**Figure 1:**
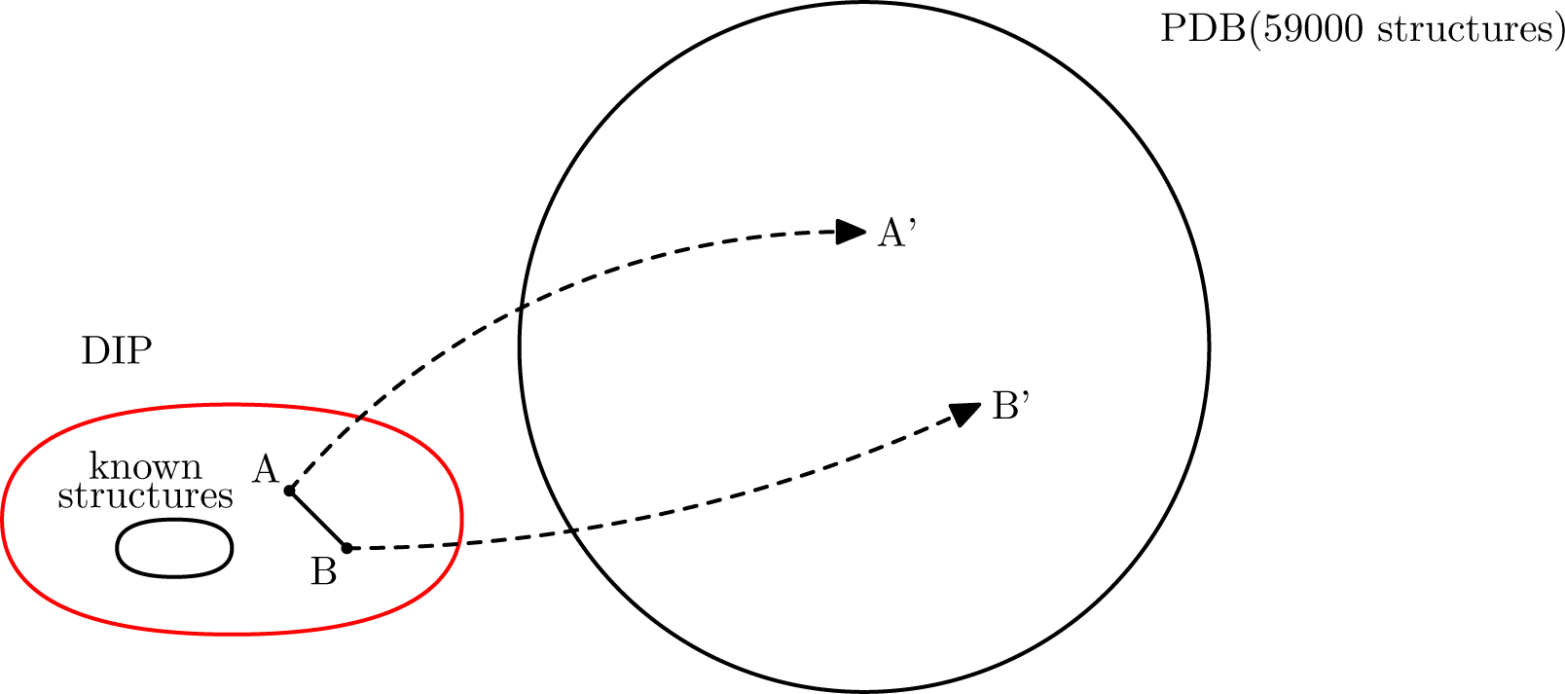
**Benchmark generation**. For each interacting pair of proteins with unknown structure, we use a proxy pair of homologs with known structure.

We built a benchmark of interacting pairs of protein structures, based on the Database of Interacting Proteins (DIP) [25] downloaded on January 26, 2009. The complete database contains 88,618 interactions among 27,496 proteins. From these interactions, we selected two subsets. The first is the "Core" dataset, which consists of a set of 4,553 interactions among 2,423 *S. cerevisiae *proteins. These interactions are considered reliable based on expression data and the presence of paralogous interacting protein pairs [1]. The second data set, "small-scale," consists of interactions that were verified by small-scale experimental methods, using techniques that reliably indicate direct physical interaction of proteins. These methods include immunoprecipitation, cross-linking, *in vitro *binding, X-ray diffraction, competition binding, electron microscopy and X-ray crystallography, for a total of 2,622 interactions among 2,408 proteins. For each of the two sets of interactions, we collected a set of protein structures using the following protocol:

1. We use PSI-BLAST to search for homologous proteins with known structures, as shown in Figure 1. For each protein in a given DIP data set, we search the domains of the PDB using PSI-BLAST for a maximum of 5 iterations [26]. For each protein, we identify at most one homolog that is longer than 50 amino acids and achieves an E-value less than 10^-20^.

2. All of our methods require negative examples for training. To avoid biasing the selection of negatives, we choose non-interacting protein pairs at random from a non-redundant subset of the PDB [27]. This subset consists of 8,261 structures, selected using the PISCES Database with a percent identify cutoff of 70% and a resolution of 2.0 Å or better (downloaded on March 2nd, 2009). At this stage, we select five times as many negative pairs as positive pairs.

3. We filter the combined collection of positive and negative pairs to remove redundant pairs. For each data set, we cluster the protein sequences using BLASTClust [26] with a threshold of 40% sequence identity. We search for cases in which pairs of proteins link the same two clusters. If all pairs are negative, then we remove all except one randomly selected pair. If one pair is positive and others are negative, then we remove the negative pairs. If two or more pairs are positive, then we remove all the pairs except the positive one that has the minimal number of (positive) neighbors.

4. Finally, we remove all negative pairs that involve a protein shorter than 50 amino acids and then randomly downsample the remaining negative pairs so that the ratio of negative to positive pairs is 3:1.

This procedure generated two different data sets. The "core" data set contains 6,175 proteins, with 1,581 interacting pairs involving 824 proteins and 4,743 non-interacting pairs. The "small-scale" set contains 6,187 proteins, with 1,392 interacting pairs involving 1,175 proteins and 4,176 non-interacting pairs. We also build a subset of the "core", called "core-subset," that contains the same number of interacting pairs as the small-scale. Note that we also generated three additional "core" and three additional "small-scale" data sets, using homology thresholds of 10^-20^and 10^-5 ^and redundancy thresholds of 40% and 90%. The sizes and cross-validation results from those data sets are summarized in the Additional file 1.

All data sets used in this study are available at http://noble.gs.washington.edu/proj/pips.

## Results

For each of the eight classifiers, we performed 5-fold cross-validation, repeated three times (3×5cv). During each SVM training, an internal 5-fold cross-validation was performed to select two regularization parameters using grid search. We selected the regularization parameter associated with interacting pairs (*C*^+^) from {10^-8^, 10^-7^,..., 10^8^}, and we selected the ratio *C*^+^/*C*^- ^from {3, 10, 100}. The parameter *k *of NN was selected from {1, 2, 3, 5, 10, 15}.

In each testing phase of the cross-validation, the proportion of negative examples with respect to positive examples is equal to *r *= 3. In a more realistic setting, this ratio would be much higher. Therefore, we extrapolate from the measured precision *p *to estimate the precision of each classifier in the case where all negative examples were considered in the benchmark. If *v *is the number of proteins, and *e *is the number of interacting pairs, then the ratio of negative to positive examples is *r' *= (v * (*v *- 1)/2 - *e*)/*e*. The estimated precision thus becomes *p*/(*p *+ *r'*/r * (1 - *p*)).

**Figure 2:**
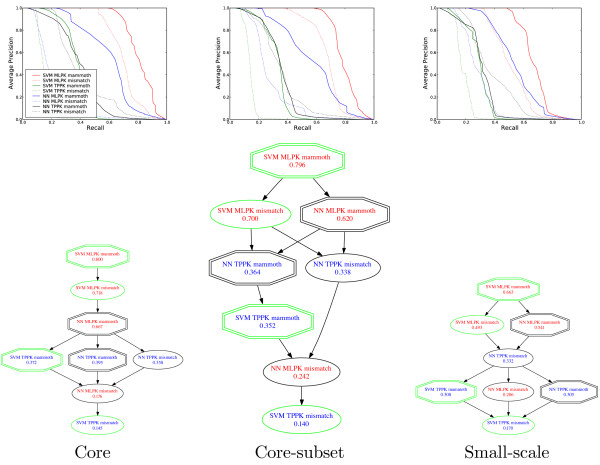
**Comparison of methods for predicting protein-protein interactions**. The top three panels plot the average precision (TP/(TP+FP)) as a function of recall (TP/(TP+FN)) for the core, core subset and small-scale benchmarks. Each precision is averaged across the 15 splits of 3×5cv, and estimated for test sets for which the negative examples are not downsampled. In the lower three panels, an edge from method *A *to *B *indicates that method *A *outperforms method *B *at *p *> 0.5 according to a Wilcoxon signed rank test applied to the area under the precision-recall curve, computed separately for each of the 15 splits of 3×5cv. Redundant edges have been removed for clarity; i.e., the figure shows the transitive reduction of the full graph.

Figure 2 shows, in the top row, precision-recall curves comparing the performance of the eight different classifiers across the 15 splits, incorporating the post-processing of precisions described in the previous paragraph. We use precision-recall curves rather than receiver operating characteristic curves because the latter removes the effect of the skewed positive-to-negative ratio [28]. In Figure 2, the performance of a random classifier would be represented by a horizontal line corresponding to a constant precision, the ratio of the positive to negative class over the whole test set (approximately *y *= 0.001). Except at high recall, all of the methods perform much better than chance.

To evaluate the statistical significance of the differences in performance observed in the top row of Figure 2, we performed Wilcoxon signed-rank tests on the area under the precision-recall curve across the 15 splits of the 3×5cv of the four data sets. The bottom row of panels in Figure 2 shows the results of this test. In the graph, an edge from method *A *to *B *indicates that method *A *outperforms method *B *according to the Wilcoxon test with *p *< 0.05. Redundant edges have been removed for clarity; i.e., the figure shows the transitive reduction of the full graph.

The signed-rank results show two expected trends across the three benchmarks. First, in general, structure-based methods perform better than the corresponding sequence-based methods. In particular, classifiers that use a MAMMOTH kernel outperform the corresponding mismatch kernel method in 9 cases out of 12, and never perform worse.

Second, SVM-based methods generally perform better than nearest neighbors methods, although this trend is not as consistent (6 wins, 2 draws and 4 losses). The relative performance of the SVM and NN classifiers seems to depend in part upon which pairwise transformation is employed. In conjunction with the MLPK transformation, the SVM outperforms the NN in all six cases. In contrast, when the TPPK transformation is employed, NN performs better than the SVM in four cases, and ties in the remaining two. Although the relative performance of the MLPK and TPPK transformations is difficult to explain [16], one may speculate that the TPPK definition (5) results in a more natural measure of similarity between pairs than the MLPK one (6); hence, TPPK is likely to behave better with a NN classifier. On the other hand, the MLPK transform can be justified from the point of view of metric learning when used in combination with an SVM [16], but MLPK remains an obscure measure of similarity between pairs, which may therefore not be optimally used by a NN classifier.

Finally, with respect to MLPK versus TPPK, the MLPK transformation performs best overall. In 9 out of 12 cases, the MLPK method outperforms the corresponding TPPK method. The only three exceptions are NN classifiers using sequence kernels, which are relatively poorly performing methods overall. Overall, our experiments suggest that, for the prediction of protein-protein interactions from structure, the MLPK SVM combined with MAMMOTH is the best method among the eight that we considered. This method performs best on all three benchmarks.

Having selected the best performing method, we used an SVM to predict novel interactions among all pairs of proteins in a non-redundant subset of the PDB. We used the PISCES Database with a percent identify cutoff of 90% and a resolution of 2.5 Å or better (downloaded on January 26, 2009), eliminating proteins shorter than 60 amino acids and longer than 300 amino acids. This set contains 9,574 structures. Prior to the SVM analysis, we eliminated pairs that are similar to any member of the training set. Specifically, for a candidate pair (*p*_1_, *p*_2_), we define the distance to a training set pair (*q*_1_, *q*_2_) as

where *E*(*p, q*) is the BLAST E-value assigned to query *p *against target *q*. We only consider candidate pairs for which the minimum distance to any member of the training set exceeds an E-value of 0.01. This filtering reduces the complete set from 45,835,525 to 45,676,254 pairs. We applied to each of these pairs the SVM MLPK MAMMOTH method, trained on 90% of the small-scale DIP benchmark, and we used the held-out 10% of the data to convert the SVM discriminants to probabilities [29].

At a threshold of 99%, the SVM predicts 4,826 novel interactions; at 90%, the SVM predicts 108,491 pairs. The latter set is publicly available via the Yeast Resource Center Public Data Repository http://yeastrc.org/pdr[30].

## Discussion

We presented eight machine learning methods for large-scale prediction of interactions between pairs of protein structures. The methods use either a NN or a SVM classifier, coupled with two kernels on protein sequences or structures, coupled with two methods for representing similarities between pairs of protein. In our cross-validation experiment, we observed--not surprisingly--that structure-based methods are generally more accurate than the methods based on sequence. Among structure-based classifiers, the SVM with an MLPK kernel performs best.

The classifiers described here only use structural or sequence information about the proteins. Further improvements in prediction accuracy could probably be obtained by taking into account other information such as expression profiles or sub-cellular localization. The integration of such heterogeneous data could for example be carried out by creating different kernels from each type of information, and merging the information by forming a linear combination of all individual kernels [9,31].

One shortcoming of our approach is that each protein interaction is predicted independently. We believe that taking into account the topology of the whole interaction graph might improve the accuracy of the predictions. However, generalizing binary prediction to more complex prediction, such as graph prediction, remains a challenging issue and an active research topic in the machine learning community.

In the course of this study we generated eight benchmarks that we make publicly available and which can be used to assess the performance of other methods for the automated prediction of interaction between protein structures. The benchmark generation depends on two parameters: the homology threshold, to be input to PSI-BLAST to find a proxy pair, and the redundancy threshold to be input to BLASTClust. For each of the benchmarks, we set the values of the homology threshold to 10^-5^, and 10^-20^, and the values of the redundancy thresholds to 40 and 90, leading to four datasets for each benchmark. Here, we reported the cross-validation results of only one dataset; however, the conclusions do not change when we consider the cross-validation experiments on the other benchmark data sets.

Finally, as pointed out in the introduction, the main motivation behind this work is to perform a large-scale screening of pairs of proteins likely to interact, before validating the prediction by more expensive computational methods such as docking, or experimental methods for pairs of particular interest. This will be the subject of future work.

## Conclusions

The eight presented machine learning methods based on kernel methods to predict the binary interaction between protein structures were evaluated on a data set of reliable interations. We draw three main conclusions from our experiments. First, using structure we can more accurately predict interactions than using sequence alone. Second, SVM methods compare favorably against the methods based on the nearest neighbor classifier. Third, the best choice of pairwise metric associated with the predictor is MLPK rather than TPPK.

## Authors' contributions

MH, JPV and WSN designed the study and drafted the manuscript. MH performed the experiments. MR included the predicted interactions in the YRC database. MH, MR, JPV and WSN read and approved the final manuscript.

## Supplementary Material

Additional file 1 **Cross-validation results on additional Core and Small-scale datasets**. The file contains the size and cross-validation results of eight datasets, including three additional Core and three additional Small-scale. The Core and Small-scale datasets are parametrized by two parameters during generation, the homology threshold and the redundancy threshold. The datasets correspond to two values for the homology threshold and two values of redundancy threshold. Click here for file
