# Supplementary material for: Large-scale prediction of protein-protein interactions from structures
Source: BMC Bioinformatics. 2010 Mar 18;11:144. doi: 10.1186/1471-2105-11-144 (PMC2845582; doi:10.1186/1471-2105-11-144)
Supplement: Additional file 1 — Cross-validation results on additional Core and Small-scale datasets. The file contains the size and cross-validation results of eight datasets, including three additional Core and three additional Small-scale. The Core and Small-scale datasets are parametrized by two parameters during generation, the homology threshold and the redundancy threshold. The datasets correspond to two values for the homology threshold and two values of redundancy threshold. [file 1471-2105-11-144-S1.PDF]

Supplement to "Large-scale prediction of protein-protein interactions from structures"

Martial Hue [1,2,3], Michael Riffle [4,5], Jean-Philippe Vert [1,2,3]
and William Stafford Noble [5,6]

August 11, 2009

[1]Mines ParisTech, Centre for Computational Biology, 35 rue Saint-Honoré, F-77305 Fontainebleau, France, [2]Institut Curie and [3]INSERM U900, F-75248, Paris, France, [4]Department of Biochemistry, University of Washington, Seattle, WA, USA, [5]Department of Genome Sciences, University of Washington, Seattle, WA, USA, [6]Department of Computer Science and Engineering, University of Washington, Seattle, WA, USA

DIP	Homology	Redundancy	Proteins	Pairs	Interacting Proteins	Interacting Pairs
core	10^{-20}	40%	6373	8788	828	2197
core	10^{-20}	90%	6422	9012	833	2253
core	10^{-5}	40%	6409	8908	841	2227
core	10^{-5}	90%	6450	9136	846	2284
small-scale	10^{-20}	40%	5877	5800	1144	1450
small-scale	10^{-20}	90%	5960	5964	1165	1491
small-scale	10^{-5}	40%	5970	5988	1167	1497
small-scale	10^{-5}	90%	6052	6156	1189	1539

Table 1: **Number of proteins and interactions in the DIP benchmarks.** The data sets of interactions between structures are parametrized by two parameters during the generation, the threshold to define a homolog pair with known structure and the threshold to reduce redundancy. We provide four "small-scale" and four "core" data sets, corresponding to the crossing of two homology thresholds and two redundancy thresholds. The table lists all four possible choices of homology and redundancy thresholds. The eight benchmarks were used for statistical comparison between classification methods, and the detail average Precision-Recall curves on all benchmarks are presented in Figure 1 and 3.

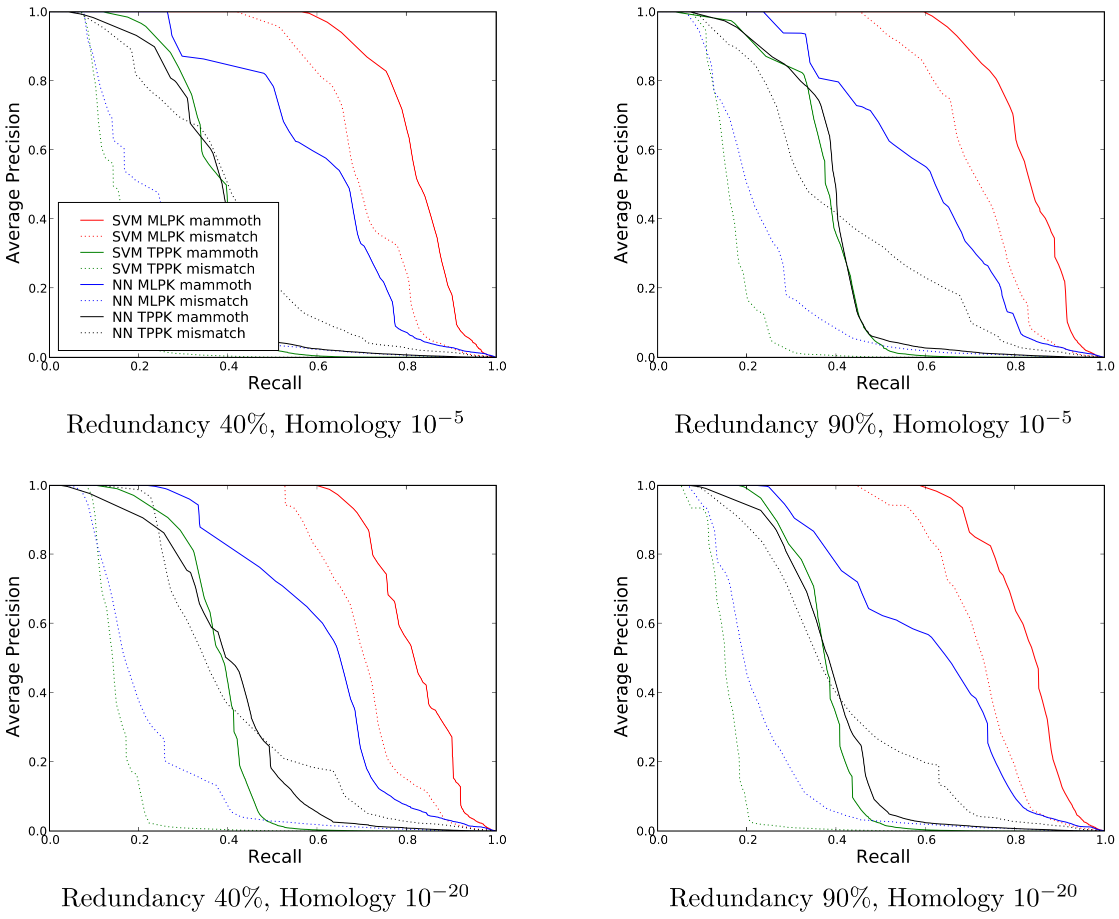

Figure 1: **Cross-validated precision-recall curves on DIP Core.** Each panel plots the average precision (TP/(TP+FP)) as a function of recall (TP/(TP+FN)). Each precision is averaged across the 15 splits of the 3x5cv, and estimated with the actual proportion of negative to positive examples.

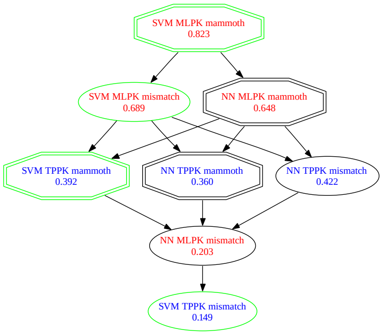

Redundancy 40%, Homology 10^{-5}

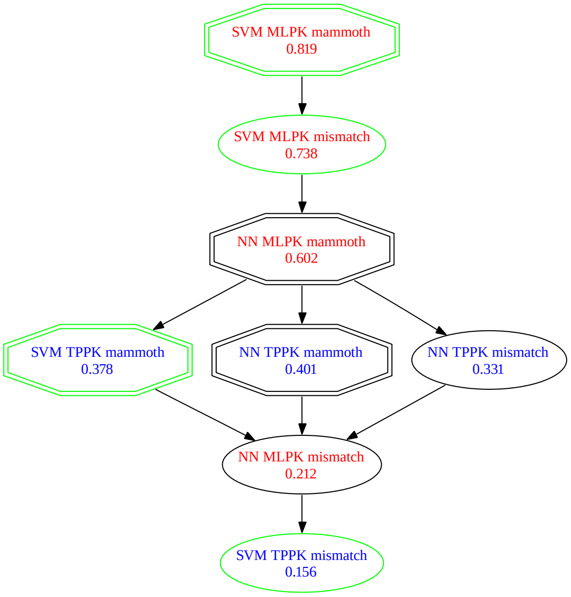

Redundancy 90%, Homology 10^{-5}

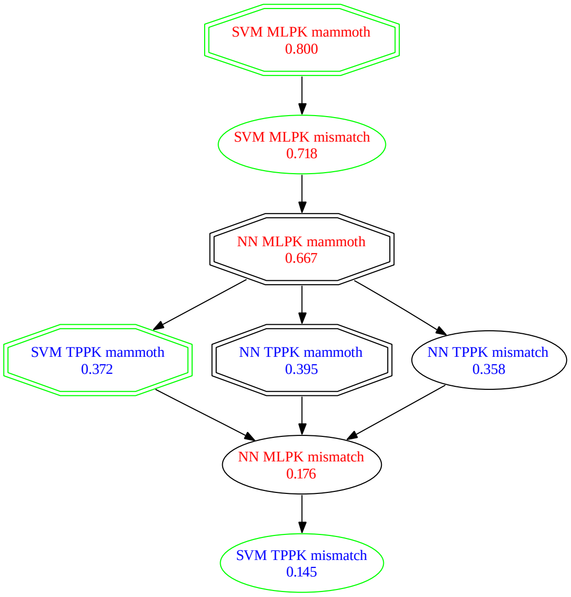

Redundancy 40%, Homology 10^{-20}

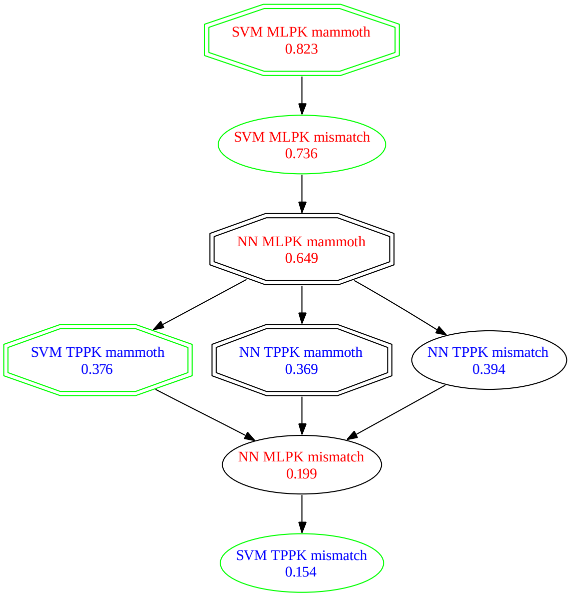

Redundancy 90%, Homology 10^{-20}

Figure 2: **Statistical ranking of prediction methods on DIP Core.** In the graph, an edge from method A to B indicates that method A outperforms method B at $p > 0.5$ according to a Wilcoxon signed rank test applied to the area under the precision-recall curve, computed separately for each of the 15 splits of 3x5cv. Redundant edges have been removed for clarity; i.e., the figure shows the transitive reduction of the full graph.

signed-ranks	SVM MLPK mammoth	SVM TPPK mammoth	NN MLPK mammoth	NN TPPK mammoth	SVM MLPK mismatch	SVM TPPK mismatch	NN MLPK mismatch	NN TPPK mismatch
SVM MLPK mammoth	—	0.0001	0.0001	0.0001	0.0001	0.0001	0.0001	0.0001
SVM MLPK mismatch		—	0.0001	0.0001	0.00209124	0.0001	0.0001	0.0001
SVM TPPK mammoth			—	0.0001		0.0001		
SVM TPPK mismatch				—				
NN MLPK mammoth			0.0001	0.0001	—	0.0001	0.0001	0.0001
NN MLPK mismatch				0.000213687		—		
NN TPPK mammoth			0.148972	0.0001		0.0001	—	0.489975
NN TPPK mismatch			0.264213	0.0001		0.0001		—

Table 2: **Pairwise wilcoxon signed-rank p values for the core benchmark.** A matrix of Wilcoxon signed-rank p values compares the eight methods for predicting interaction between protein structures in table 2 and 3. A significant p value indicates that the row method outperforms the column method.

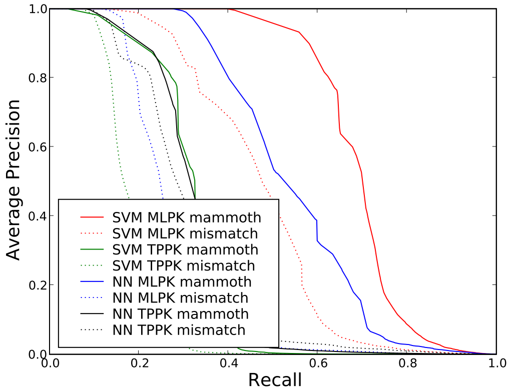

Redundancy 40%, Homology 10^{-5}

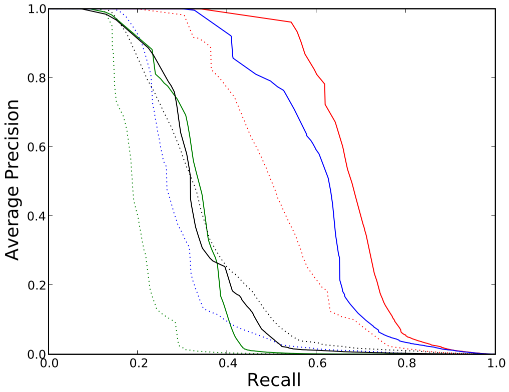

Redundancy 90%, Homology 10^{-5}

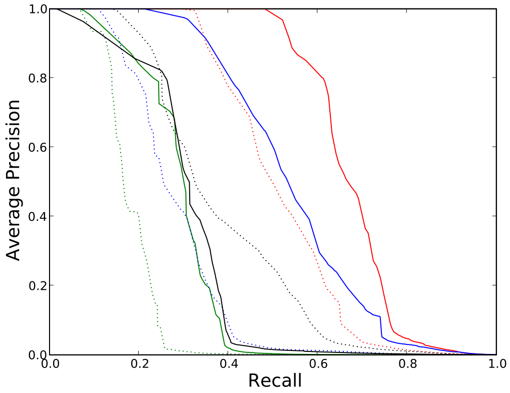

Redundancy 40%, Homology 10^{-20}

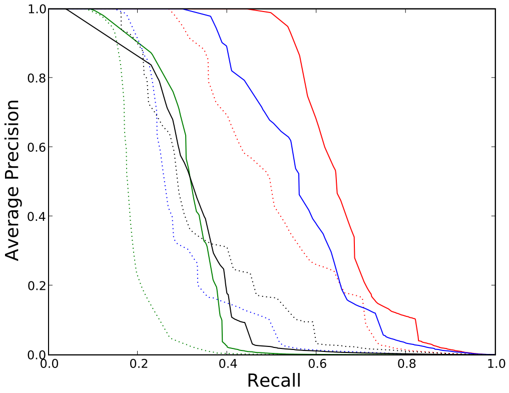

Redundancy 90%, Homology 10^{-20}

Figure 3: **Cross-validated precision-recall curves on small scale DIP.** Each panel plots the average precision (TP/(TP+FP)) as a function of recall (TP/(TP+FN)). Each precision is averaged across the 15 splits of the 3x5cv, and estimated with the actual proportion of negative to positive examples.

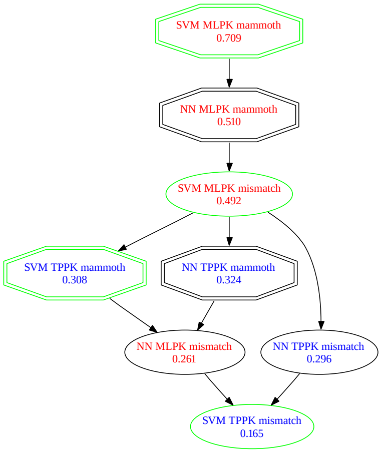

Redundancy 40%, Homology 10^{-5}

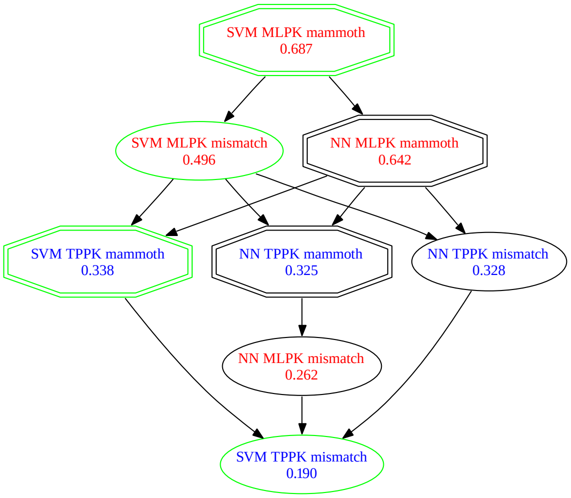

Redundancy 90%, Homology 10^{-5}

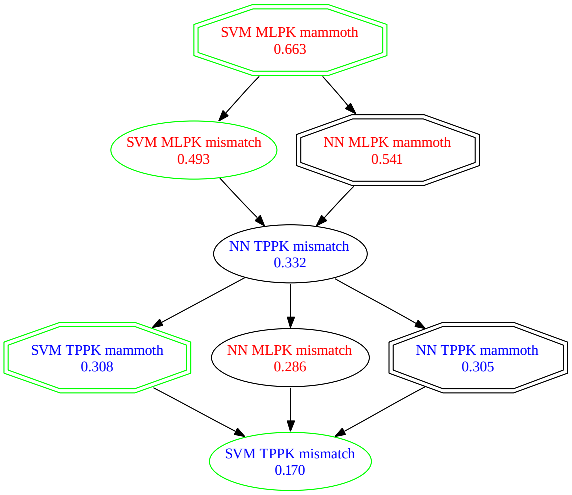

Redundancy 40%, Homology 10^{-20}

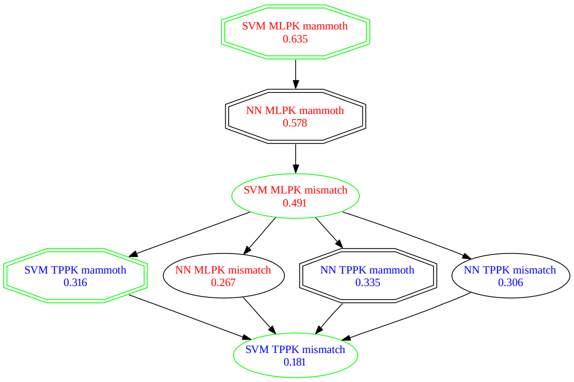

Redundancy 90%, Homology 10^{-20}

Figure 4: **Statistical ranking of prediction methods on small scale DIP.** In the graph, an edge from method A to B indicates that method A outperforms method B at $p > 0.5$ according to a Wilcoxon signed rank test applied to the area under the precision-recall curve, computed separately for each of the 15 splits of 3x5cv. Redundant edges have been removed for clarity; i.e., the figure shows the transitive reduction of the full graph.

signed-ranks	SVM MLPK mammoth	SVM TPPK mammoth	NN MLPK mammoth	NN TPPK mammoth	SVM MLPK mismatch	SVM TPPK mismatch	NN MLPK mismatch	NN TPPK mismatch
SVM MLPK mammoth	—	0.0001	0.0001	0.0001	0.000152553	0.0001	0.0001	0.0001
SVM MLPK mismatch		—	0.0001	0.0001		0.0001	0.0001	0.0001
SVM TPPK mammoth			—	0.0001		0.39098		
SVM TPPK mismatch				—				
NN MLPK mammoth		0.352748	0.0001	0.0001	—	0.0001	0.0001	0.000839252
NN MLPK mismatch				0.0001		—		
NN TPPK mammoth			0.187683	0.0001		0.264213	—	
NN TPPK mismatch			0.0193126	0.0001		0.00314482	0.0288398	—

Table 3: **Pairwise Wilcoxon signed-rank p values for the small-scale benchmark.** A matrix of Wilcoxon signed-rank p values compares the eight methods for predicting interaction between protein structures in table 2 and 3. A significant p value indicates that the row method outperforms the column method.
